# Periodontal Treatment Restores Gingival Crevicular Fluid and Serum Periostin Levels

**DOI:** 10.1111/jcpe.70206

**Published:** 2026-10-11

**Authors:** Aysegul Sarı, Oguzhan Ozcan, Filiz Kaçmaz, Anton Sculean, Alpdogan Kantarci

**Affiliations:** ^1^ Department of Periodontology, Faculty of Dentistry Hatay Mustafa Kemal University Hatay Turkey; ^2^ Periodontology Unit, Centre for Host‐Microbiome Interactions King's College London Dental Institute London UK; ^3^ Department of Biochemistry, Faculty of Medicine Mustafa Kemal University Hatay Turkey; ^4^ Department of Molecular Biochemistry and Genetics Health Science Institute, Mustafa Kemal University Hatay Turkey; ^5^ Department of Periodontology, School of Dental Medicine University of Bern Bern Switzerland; ^6^ School of Dentistry University of Minnesota Minneapolis Minnesota USA

**Keywords:** gingival crevicular fluid, periodontal treatment, periodontitis, periostin, serum

## Abstract

**Background and Aim:**

To investigate the effect of periodontal treatment on periodontal inflammation and periostin levels in patients with severe periodontitis.

**Methods:**

This study included two groups (periodontitis and periodontally healthy), each comprising 24 individuals. Periodontal clinical parameters were recorded, and serum samples were collected at baseline and on Days 30 and 90 following non‐surgical periodontal treatment (NSPT). Gingival crevicular fluid (GCF) samples were collected at baseline and on Days 1, 2, 14, 30 and 90 following NSPT. Periostin, interleukin‐1 beta (IL‐1β) and fibroblast growth factor‐2 (FGF‐2) levels were assessed in serum and GCF samples.

**Results:**

Clinical periodontal parameters decreased from baseline to Days 30 and 90 after NSPT (*p* = 0.001). Patients with periodontitis had higher levels of IL‐1β in serum (*p* = 0.020) and GCF (*p* = 0.001) than healthy individuals. Following periodontal treatment, serum IL‐1β levels decreased at Days 30 (*p* = 0.036) and 90 (*p* = 0.025) while GCF IL‐1β levels showed a reduction at Day 14 (*p* = 0.001). Periostin levels in GCF in patients with periodontitis were lower than in healthy individuals at baseline (*p* = 0.001). It increased after treatment, reaching peak values on Day 14 (*p* = 0.001), followed by a gradual decrease on Days 30 (*p* = 0.007) and 90 (*p* = 0.001). Levels of FGF‐2 in GCF increased after periodontal treatment compared with baseline in patients with periodontitis, reaching peak levels on Day 2 (*p* = 0.001). Following periodontal treatment, periostin and FGF‐2 levels remained higher in the periodontitis group than in the healthy group (*p* < 0.05), whereas IL‐1β levels decreased to levels comparable to those of the healthy group by Day 30 (*p* ≥ 0.05).

**Conclusion:**

In parallel with the reduction in periodontal tissue inflammation and IL‐1β, periostin levels in GCF increased in response to periodontal treatment, suggesting its involvement in tissue healing in patients with periodontitis.

## 1 Introduction

The periodontium is a highly adaptive and dynamic tissue complex capable of sustaining diverse microbiological, inflammatory and mechanical conditions through intricate molecular mechanisms that involve growth factors, transcriptional regulators and extracellular matrix (ECM) components (Bartold and Narayanan 2006). The periodontal ligament is a key structure in maintaining, repairing and regenerating periodontal tissues (Nuñez et al. 2019). Analysis of gingival crevicular fluid (GCF) following non‐surgical periodontal therapy (NSPT) enables assessment of mediators involved in inflammation, tissue repair and regeneration (Buduneli et al. 2024; Koidou et al. 2020). Monitoring the early phases of healing is particularly important, as inflammatory and granulation tissue responses generally occur within the first 2 weeks post treatment, while longer term changes can be evaluated over 3 months (Koidou et al. 2020). Systemic effects of periodontitis can also be examined by measuring serum mediator levels (Ahmad et al. 2025; Sari et al. 2021).

Basic fibroblast growth factor (bFGF/FGF‐2), belonging to the heparin‐binding growth factor family, supports early wound healing and suppresses their differentiation into mineralised tissue‐forming cells, potentially contributing to periodontal regeneration (Murakami et al. 1999). In addition, FGF‐2 may be involved in the regulation of hyaluronan production, suggesting a role in both periodontal tissue homeostasis and regeneration (Shimabukuro et al. 2005). Periostin (POSTN) is a matricellular protein comprising 811 amino acids that functions as a non‐structural component of the ECM (Kruzynska‐Frejtag et al. 2004). It contributes to cell activation across multiple biological processes, including cell migration, wound repair and tooth development (Hamilton 2008). Periostin helps in preserving the structural integrity of connective tissues by providing a stable microenvironment (Hamilton 2008).

The literature contains conflicting results regarding periostin levels and its role in periodontal inflammatory or regenerative processes (Abdolalian et al. 2023; Sari et al. 2022; Teles et al. 2025). One meta‐analysis reported that periostin levels decrease in the presence of periodontitis (Abdolalian et al. 2023), while other studies reported increased levels in the presence of periodontal disease (Arslan et al. 2021; Sari et al. 2022). One study reported decreased periostin levels after NSPT (Arslan et al. 2021), whereas others reported increased levels at different time points during the early healing phase (Al‐Rihaymee and Sh Mahmood 2023; Kumaresan et al. 2016). A recent study showed that periostin levels were higher at stable sites in patients with periodontitis (Teles et al. 2025). Thus, the involvement of periostin in the inflammatory and repair/regenerative phases of periodontitis, as well as its variation following NSPT, remains unclear. Addressing this question, especially during the early healing period after NSPT, requires clinical investigations with follow‐up assessments. Accordingly, the present study aimed to assess periostin concentrations in GCF and serum during the initial phase of healing and to compare findings between periodontitis patients and periodontally healthy individuals. A secondary objective was to explore the associations between periostin and other inflammatory and regenerative mediators. The null hypothesis proposed was that periostin expression does not change across early healing time points after NSPT in patients with periodontitis and does not differ between patients with periodontitis and individuals with a healthy periodontium at each time point.

## 2 Materials and Methods

The prospective cohort study was designed to characterise periostin changes over time after NSPT in serum and GCF samples and was carried out at the Department of Periodontology, Faculty of Dentistry, Hatay Mustafa Kemal University, Hatay, Turkey. The Ethics Committee approved the study protocol for the Use of Human Subjects in Research of Hatay Mustafa Kemal University (Protocol No: 2019/118). The study was carried out in accordance with the tenets of the Declaration of Helsinki and the CONSORT guidelines (Schulz et al. 2010). The individuals enrolled in the study were registered for the clinical trial (ClinicalTrials.gov ID: NCT05535049). Participants were recruited from December 2019 to August 2021. Written informed consent was obtained from each participant before the clinical periodontal examination.

### 2.1 Study Population

Forty‐eight adults were selected for the study and allocated to two groups of 24, matched by sex: periodontally healthy and periodontitis. The periodontally healthy group participants were chosen to match the demographic profile of the periodontitis group. Inclusion criteria were (a) never smokers, (b) no history of periodontal therapy within the previous 6 months, (c) no antibiotic use within the past 3 months and (d) the presence of more than 18 teeth. Exclusion criteria were (a) pregnancy, (b) current or former smoking and (c) any self‐reported systemic illness or condition that could confound outcomes—such as AIDS, rheumatoid arthritis, cardiovascular disorders or diabetes.

### 2.2 Clinical Examination

Periodontal clinical parameters were recorded by a single calibrated examiner (A.Sa.). The intra‐class correlation coefficient was 0.95. Intra‐examiner reproducibility for clinical attachment level (CAL) was assessed by repeated examinations of 10 subjects at 1‐h intervals. Clinical periodontal measurements were assessed using the following parameters in both groups and on Days 30 and 90 after NSPT in the periodontitis group using a millimetre‐marked Williams periodontal probe (Hu‐Friedy, Chicago, IL, USA). Probing pocket depth (PPD), CAL, plaque index (PI) (Silness and Loe 1964), gingival index (GI) (Loe and Silness 1963) and percentage bleeding on probing (BOP) (Ainamo and Bay 1975) were recorded at six sites per tooth (mesio‐buccal, buccal, disto‐buccal, mesio‐lingual, lingual and disto‐lingual).

Diagnosis of periodontal diseases and conditions was made according to the radiographic and clinical diagnostic criteria proposed by the 2017 World Workshop on Classification of Periodontal and Peri‐implant Diseases and Conditions (Caton et al. 2018). Individuals with a BOP < 10% (without attachment loss and radiographic bone loss) were considered to have periodontal health (Tonetti and Sanz 2019). The criteria for periodontitis included patients with CAL ≥ 5 mm in two or more interproximal sites and PPD ≥ 6 mm in one or more interproximal sites. Only Stage III–IV (severe) periodontitis was included in the present study (Tonetti et al. 2018).

### 2.3 Periodontal Intervention

Periodontitis patients were treated by one‐stage full‐mouth disinfection, which included full‐mouth scaling and root planing procedures performed over two visits within a 24‐h period as proposed, under local anaesthesia (lidocaine 8 mL, 2% with epinephrine 1:80,000) (Quirynen et al. 1995). Each session lasted 60 min and was carried out on two consecutive days. Subgingival irrigation and tongue brushing were performed with 1% CHX gel for 1 min. Mouthwashes with 0.12% CHX were performed for 30 s at the beginning and end of each session, and twice daily for 2 weeks (Quirynen et al. 1995).

### 2.4 GCF Sample Collection

GCF samples were collected 8 h after night fasting and 1 week after baseline clinical periodontal measurements in the two groups, and then on Days 1, 2, 14, 30 and 90 after NSPT (Akalin et al. 2007). The samples were collected from sites with PPD ≥ 6 mm and positive BOP in patients with periodontitis. Six GCF samples were taken per participant. Samples were collected from a mesial and a distal site on each of the three teeth (incisors, premolars and molars) in the different quadrants. At each follow‐up appointment, samples were obtained from the same teeth/sites sampled at baseline. Saliva contamination was prevented by cotton‐roll isolation and gentle air‐drying. Samples contaminated with saliva or blood were excluded. After supragingival plaque removal with a periodontal curette, GCF samples were collected within 30 s using Periopaper strips (Periopaper; Oraflow Inc., Plainview, NY) by the orifice method (Rüdin et al. 1970). GCF volumes were measured on a pre‐calibrated electronic device (Oraflow Inc., Plainview, NY) (Chapple et al. 1999) and converted to microlitres (μL) according to a standard curve. All strips were pooled into Eppendorf microcentrifuge tubes and stored at −80°C until biochemical analysis.

### 2.5 Serum Sample Collection

Peripheral venous blood samples were taken from the patients. The serum was separated from the cells by centrifugation at 2000 rpm for 10 min, and the supernatant was stored at −80°C until biochemical analysis.

### 2.6 Laboratory Analyses

All GCF samples were thawed, and 375 μL of phosphate‐buffered saline (pH: 7.00, 10 mM Na_2_PO_4_, 137 mM NaCl and 2.7 mM KCl) was added to the Eppendorf tubes containing the sample strips. Subsequently, samples were eluted for 30 min at room temperature and centrifuged at 11,000*g* for 15 min at 4°C (Chapple et al. 1997). Periostin, IL‐1β and FGF‐2 levels in GCF and serum were quantified by ELISA (450 nm) using a Thermo Fisher Scientific Multiskan Go reader (Finland) with commercially available kits. Periostin was measured using ELABSCIENCE Human POSTN/OSF‐2 (Cat No: E‐EL‐H6113; range: 3.13–200 ng/mL; sensitivity: 1.88 ng/mL; intra‐/inter‐assay CV < 10%). IL‐1β was measured using ELABSCIENCE Human IL‐1β (Cat No: E‐EL‐H0149; range: 7.81–500 pg/mL; sensitivity: 4.69 pg/mL; CV < 10%). FGF‐2 was measured using ELABSCIENCE Human FGF21 (Cat No: E‐EL‐H0074; range: 31.25–2000 pg/mL; sensitivity: 18.75 pg/mL; CV < 10%).

### 2.7 Statistical Analysis/Power Calculation

The primary outcome of the study was the differences in periostin levels (ng/30 s) in GCF across multiple time intervals after periodontal treatment. To find a significant difference in periostin levels (ng/30 s) in GCF in the periodontitis group at different post‐periodontal treatment terms (six time points) with a large effect size (Cohen's *d* = 1.00), the minimum required sample size was estimated as 22 for each group (*α* = 0.05; 1 − β = 0.90). GPower package version 3.1 was used for sample size calculations. The normality of continuous variable distributions was tested using the Shapiro–Wilk test. Descriptive statistics were presented as mean and standard deviation (mean ± SD) for parametrically distributed variables and as median (interquartile range, IQR) for the non‐parametrically distributed variables. The Chi‐square test was used to analyse the associations between categorical variables. Student's *t*‐test for parametric data and the Mann–Whitney *U* test for non‐parametric data were used to compare two independent groups. The Friedman test was performed for non‐parametric dependent variables at different time points. Post hoc pairwise comparisons were conducted using the Wilcoxon signed‐rank test with Bonferroni correction for multiple comparisons. Statistical analysis was performed with SPSS for Windows, version 24.0, and a *p*‐value < 0.05 was considered statistically significant.

## 3 Results

### 3.1 Demographic Variables

The demographic characteristics of the groups are shown in Table 1. Sex, age and body mass index (BMI) were not different between the groups (*p* = 1, *p* = 0.702 and *p* = 0.264, respectively).

**TABLE 1:** Characteristics of the study population.

Variables	Periodontally healthy group (*n* = 24)	Periodontitis group (*n* = 24)	*p*
Sex			
Male	11 (45.8%)	11 (45.8%)	1.000
Female	13 (54.2%)	13 (54.2%)	
Age	40.54 ± 9.07	41.54 ± 8.91	0.702
BMI	23.06 ± 2.31	23.85 ± 3.04	0.264

*Note:* *p*‐values were obtained from the χ^2^ test for categorical variables and from the Student's *t*‐test for a parametric variable. Data are expressed as median and interquartile range *n* (%) and mean ± SD. Statistically significant at *p* < 0.05.

### 3.2 Periodontal Clinical Findings

The periodontal parameters of the full mouth and the sampled teeth are presented in Figure 1. The sampled periodontal parameters and GCF values were the average of the mesial and distal sites for three teeth. At baseline, all clinical periodontal parameters of the full mouth and sampled teeth (site‐specific) were lower in the periodontally healthy group than the periodontitis group (*p* = 0.001). All clinical periodontal parameters decreased from baseline on Days 30 and 90 after NSPT (*p* = 0.001). There were no differences between PI and GI values on Days 30 and 90 of the full mouth and sampled teeth and BOP (%) values of sampled teeth (*p* ≥ 0.05). PPD and CAL values of the full mouth and sampled teeth and BOP (%) values of the full mouth decreased from Day 30 to Day 90 (*p* = 0.001). PI values were lower in the periodontally healthy group than in the periodontitis group at all time points after NSPT, while GI, BOP (%), PPD and CAL were higher (*p* < 0.05).

**FIGURE 1:**
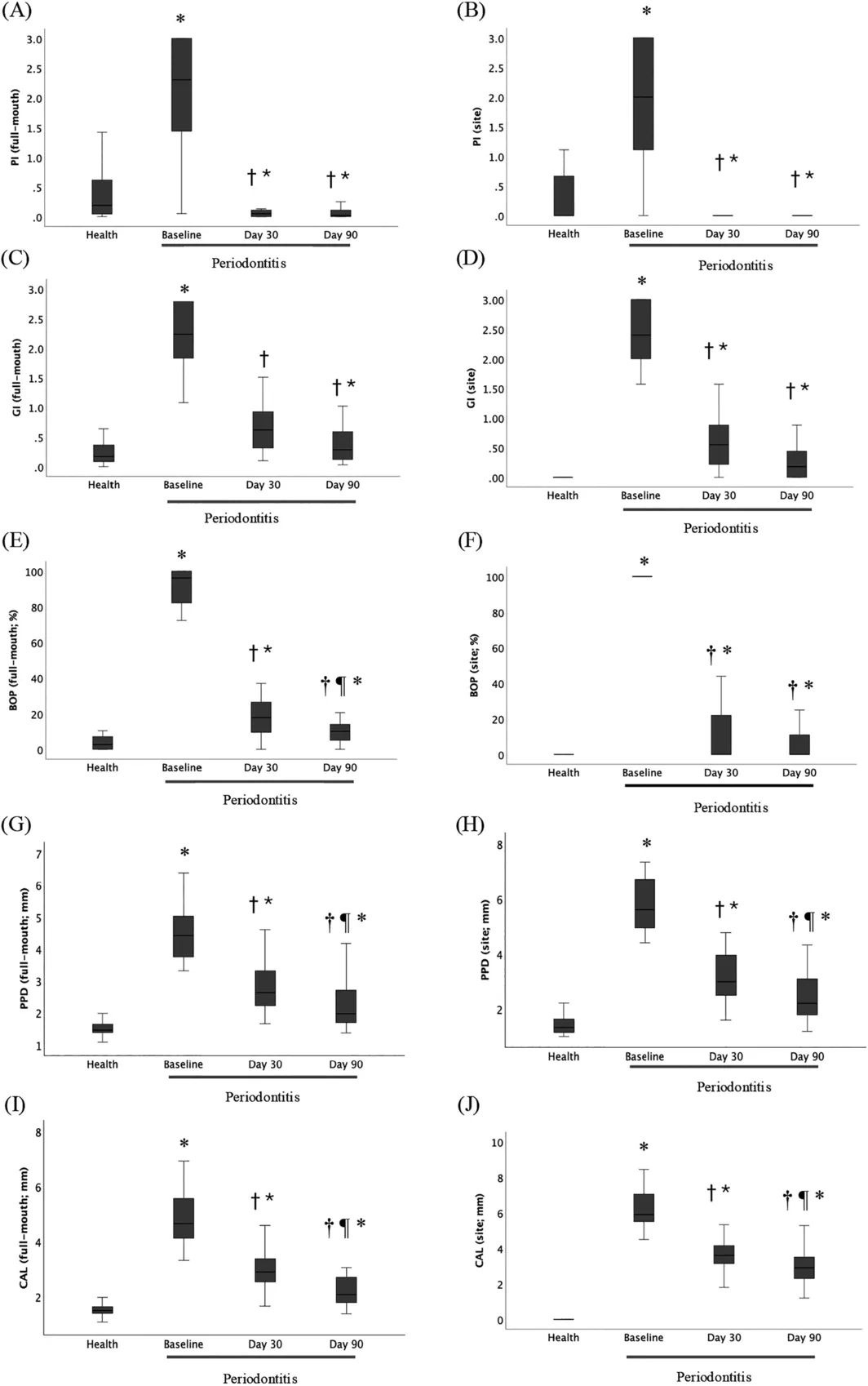
Periodontal index full‐mouth and site values between groups and among baseline and after non‐surgical periodontal treatment time points. (A) PI full‐mouth values between groups and among time points. (B) PI site‐specific values between groups and among time points. (C) GI full‐mouth values between groups and among time points. (D) GI site‐specific values between groups and among time points. (E) BOP (%) full‐mouth values between groups and among time points. (F) BOP (%) site‐specific values between groups and among time points. (G) PPD (mm) full‐mouth values between groups and among time points. (H) PPD (mm) site‐specific values between groups and among time points. (I) CAL (mm) full‐mouth values between groups and among time points. (J) CAL (mm) site‐specific values between groups and among time points. *p*‐value was obtained from the Mann–Whitney *U* test for between‐group variables and from the Friedman test for the among‐time variables. Statistically significant at *p* < 0.05. * versus health, † versus baseline in P group, ¶ versus first month in P group. GI, gingival index; PI, plaque index; PPD, probing pocket depth.

### 3.3 Biochemical Findings

#### 3.3.1 GCF Findings

Findings on the total amount of GCF are presented in Figure 2 (Table 2). GCF volumes (μL) in the periodontally healthy group were lower than in the periodontitis group (*p* = 0.001). In the periodontitis group, the values decreased over time, particularly from baseline to Days 2, 14, 30 and 90 (*p* = 0.001). There were no differences in GCF volumes (μL) between the periodontally healthy group at baseline and the periodontitis group on Day 90 (*p* ≥ 0.05).

**FIGURE 2:**
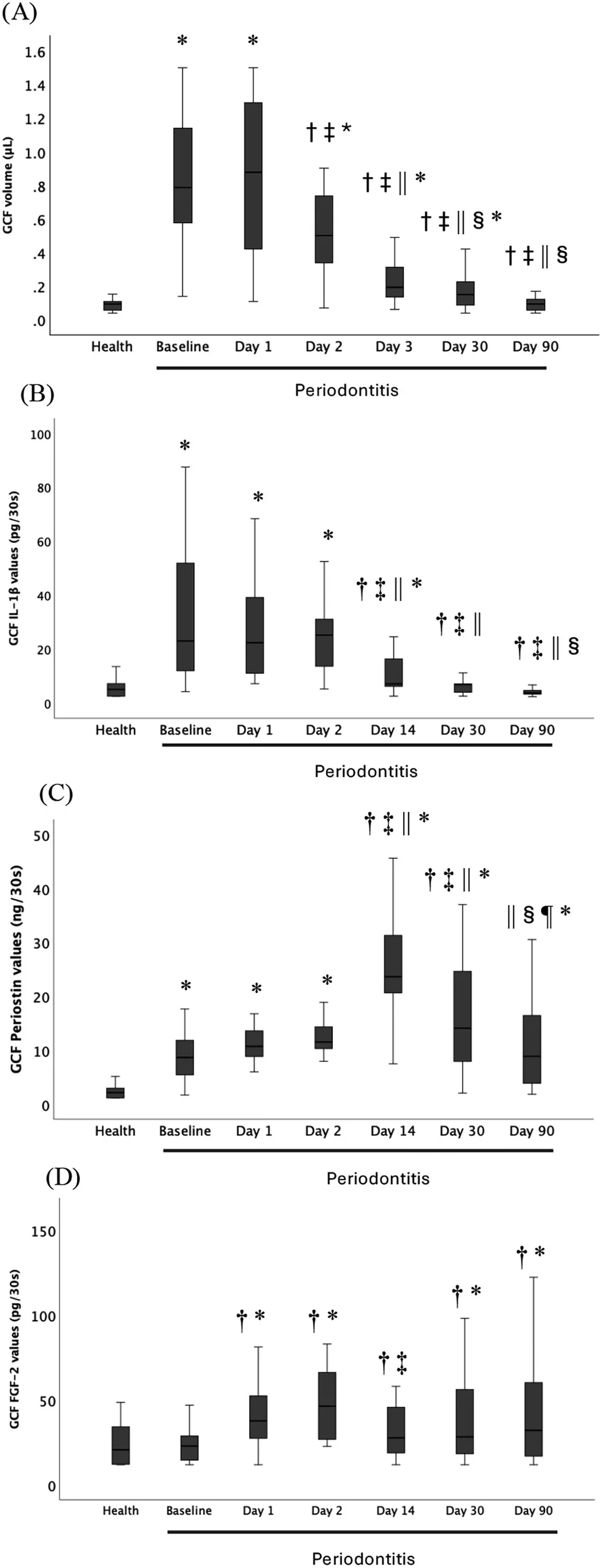
GCF volume and the biochemical parameters values between groups and among baseline and after non‐surgical periodontal treatment time points. (A) GCF volumes between groups and among time points. (B) IL‐1β values between groups and among time points. (C) Periostin values between groups and among time points. (D) FGF‐2 values between groups and among time points. *p*‐values were obtained from the Mann–Whitney *U* test for between‐group variables and from the Friedman test for the among‐time variables. Statistically significant at *p* < 0.05. * versus health, † versus baseline in P group, ‡ versus 24th h in P group, || versus 48th h in P group, § versus 14th day in P group, ¶ versus first month in P group. FGF, fibroblast growth factor; IL, interleukin.

**TABLE 2:** Comparison of GCF volume and biochemical parameters values at baseline between groups and among baseline and after non‐surgical periodontal treatment time points.

Variables	Periodontally healthy group (*n* = 24)	Periodontitis group (*n* = 24)	*p* (between‐group)
GCF (μL)			
Baseline	0.1 (0.11–0.06)	0.79 (1.14–0.58)	0.001
24 h		0.88 (1.29–0.42)	
48 h		0.5 (0.74–0.34)* † ‡	
Day 14		0.2 (0.31–0.14)* † ‡ ||	
Month 1		0.15 (0.23–0.09)† ‡ || §	
Month 3		0.1 (0.12–0.06)† ‡ || §	
*p* (among time)		0.001	
Periostin total amount (ng/30 s)			
Baseline	2.17 (1.17–2.98)	8.67 (5.47–11.85)	0.001
24 h		10.73 (8.84–13.59)* †	
48 h		11.7 (10.46–14.34)* †	
Day 14		23.65 (20.63–31.27)* † ‡ ||	
Month 1		14.09 (7.93–24.64)* † §	
Month 3		8.89 (3.9–16.44)* || § ¶	
*p* (among time)		0.001	
Periostin concentration (ng/μL)			
Baseline	9.77 (6.03–12.52)	3.46 (2.55–4.44)	0.001
24 h		4.31 (3.16–9.73)	
48 h		8.29 (5.14–11.21)†	
Day 14		39.49 (26.96–60.77)† ‡ ||	
Month 1		28.75 (18.09–51.29)† ‡ ||	
Month 3		33.64 (18.29–55.26)† ‡, ||	
*p* (among time)		0.001	
IL‐1β total amount (pg/30 s)			
Baseline	5.13 (2.63–7.31)	23.1 (12.06–51.91)	0.001
24 h		22.44 (11.12–39.2)	
48 h		25.28 (13.73–31.13)	
Day 14		7.47 (6.46–16.4)* † ‡ ||	
Month 1		6.96 (4.11–7.18)† ‡ ||	
Month 3		3.84 (3.38–4.78)† ‡ || §	
*p* (among time)		0.001	
IL‐1β concentration (pg/μL)			
Baseline	22.06 (10.8–31.22)	9.13 (5.41–18.92)	0.021
24 h		7.57 (5.6–22.28)	
48 h		13.4 (10.2–19.35)	
Day 14		15.33 (8.63–31.78)	
Mouth 1		15.72 (9.35–23.41)	
Mouth 3		15.37 (11.16–24.09)	
*p* (among time)		0.231	
FGF total amount (pg/30 s)			
Baseline	20.57 (12.06–34.06)	22.73 (14.41–28.72)	0.787
24 h		37.95 (29.81–52.3)* †	
48 h		46.27 (26.76–66.1)* †	
Day 14		27.67 (19.63–45.63)† ||	
Month 1		28.18 (18.25–56.06)* †	
Month 3		32.04 (16.85–60.11)* †	
*p* (among time)		0.001	
FGF concentration (pg/μL)			
Baseline	92.68 (52.2–139.66)	9.51 (6.79–14.73)	0001
24 h		18.03 (13.11–29.25)	
48 h		27.24 (19.95–48.14)†	
Day 14		46.33 (37.47–67.15)† ‡	
Month 1		84.77 (46.45–155.1)† ‡ ||	
Month 3		143.67 (66.56–236.29)† ‡ || §	
*p* (among time)		0.001	

*Note:* *p*‐values were obtained from the Mann–Whitney *U* test for between‐group variables and from the Friedman test for the among‐times variables. Data are expressed as median and interquartile range (IQR: 25–75). Statistically significant at *p* < 0.05. * versus health, † versus baseline, ‡ versus 24th h, || versus 48th h, § versus 14th day, ¶ versus first month.

Abbreviations: FGF, fibroblast growth factor; IL, interleukin.

The total amount of periostin and IL‐1β (pg/30 s) in GCF was lower in the periodontally healthy group than in the periodontitis group (*p* = 0.001). There were no differences between groups in the total amount of FGF‐2 (pg/30 s) levels in GCF (*p* ≥ 0.05).

The total amount of periostin (ng/30 s) in GCF gradually increased and reached a peak on Day 14 after treatment in the periodontitis group (*p* = 0.001). The level decreased from Day 14 to Days 30 and 90 (*p* = 0.007, 0.001, respectively). The total amount of IL‐1β in GCF (pg/30 s) decreased dramatically from the Day 14 after NSPT in the periodontitis group (*p* = 0.001). The total amount of FGF‐2 in GCF (pg/30 s) increased dramatically from baseline to Day 1 and reached a peak on Day 2. The level gradually decreased from Day 14 to Day 90. While there was no difference between the total amount of IL‐1β in GCF at baseline and on Days 30 and 90 (*p* ≥ 0.05), periostin and FGF‐2 levels were still higher on Days 30 and 90 compared to their baseline levels (*p* < 0.05). At all time points following periodontal treatment, except for Day 14 for FGF‐2 levels, periostin and FGF‐2 levels remained higher in the periodontitis group than in the healthy group (*p* < 0.05). By Day 30, IL‐1β levels in the periodontitis group had decreased to levels comparable to those of the periodontally healthy group.

Periostin (ng/μL), IL‐1β (pg/μL) and FGF‐2 (pg/μL) concentration levels in GCF in the periodontally healthy group were lower than in the periodontitis group (*p* = 0.021, 0.001, 0.001, respectively) (Table 2). Periostin concentration (ng/μL) levels increased from Day 2 and peaked on Day 14 after treatment in patients with periodontitis. There were no differences among Days 14, 30 and 90 (*p* ≥ 0.05). There were no differences between sampling times in IL‐1β concentration (pg/μL) levels in GCF (*p* ≥ 0.05). FGF‐2 concentration (pg/μL) levels gradually increased after Day 2 (*p* = 0.001) in patients with periodontitis. There was no difference between the levels on Days 30 and 90 (*p* = 0.440).

#### 3.3.2 Serum Findings

Serum findings are presented in Table 3. Serum IL‐1β levels (pg/mL) were lower in periodontally healthy individuals than in patients with periodontitis (*p* = 0.020). After periodontal treatment, serum IL‐1β levels (pg/mL) decreased gradually from baseline to Days 30 and 90 (*p* = 0.036, *p* = 0.025, respectively), with no difference between Day 30 and Day 90 (*p* = 0.885). There were no differences between the groups or sampling times in FGF‐2 (pg/mL) (*p* = 0.564) and periostin levels (ng/mL) in serum (*p* = 0.695).

**TABLE 3:** Serum biochemical parameters at baseline between groups and among baseline and after non‐surgical periodontal treatment time points.

Variables	Periodontally healthy group (*n* = 24)	Periodontitis group (*n* = 24)	*p* (between‐group)
IL‐1β (pg/mL)			
Baseline	17.02 (10.87–31.11)	22.69 (21.52–32.02)	0.020
Month 1		15.31 (12.51–18.45)†	
Month 3		16.89 (11.45–25.1)†	
*p*‐value (among times)		0.042	
FGF‐2 (pg/mL)			
Baseline	114.28 (9.99–37.38)	128.11 (76.39–167.14)	0.564
Month 1		149.64 (96.77–203.39)	
Month 3		41.88 (17.13–76.45)	
*p*‐value (among times)		0.420	
Periostin (ng/mL)			
Baseline	26.6 (9.99–37.38)	24.84 (10.47–55.13)	0.695
Month 1		23.15 (11.99–48.88)	
Month 3		41.88 (17.13–76.45)	
*p*‐value (among times)		0.311	

*Note:* *p*‐values were obtained from the Mann–Whitney *U* test for between‐group variables and from the Friedman test for the among‐times variables. Data are expressed as median and interquartile range (IQR: 25–75). Statistically significant at *p* < 0.05. † versus baseline.

Abbreviations: FGF‐2, fibroblast growth factor; IL, interleukin.

## 4 Discussion

The current study demonstrated that the periostin levels in GCF were higher in periodontitis. Their levels gradually increased after NSPT, reaching peak levels on Day 14 after treatment, and then gradually decreased on Days 30 and 90, refuting the null hypothesis that periostin expression followed a similar trend across time points and between diseased and healthy conditions.

Periostin does not passively track the ECM response to trauma as matrix cell molecules do (Padial‐Molina et al. 2015). In the periodontium, as in other tissues, periostin is secreted by fibroblasts specialised to regulate cellular interactions and support matrix organisation (Conway and Molkentin 2008; Padial‐Molina et al. 2015). Total periostin levels in GCF were higher, in parallel with IL‐1β, in periodontitis patients in the present study. While this finding is compatible with some previous findings (Arslan et al. 2021; Sari et al. 2025), it contradicts some others (Balli et al. 2015). The present study's findings support the view that acute exposure to pro‐inflammatory factors, such as TNF‐α and *Porphyromonas gingivalis* LPS, can trigger periostin release from gingival fibroblasts (Nakajima et al. 2014). In the gingival epithelium, periostin promotes an increased rate of proliferation and migration, similar to that observed in skin epithelial cells as a potential enhancer of wound closure after injury (Rosselli‐Murai et al. 2013). Similarly, epithelial cells, the first line of defence in periodontal tissues, increase periostin release to promote gingival sealing around the teeth in response to bacteria (Padial‐Molina et al. 2015).

The total amount of periostin in GCF increased immediately and reached a peak at Day 14 after treatment in the periodontitis group; it then decreased gradually thereafter, in parallel with FGF‐2. Their levels were similar to their baseline values on Day 90. In two previous studies with a similar prospective study design, while Al‐Rihaymee and Sh Mahmood (2023) found increased periostin levels in GCF on post‐op Days 30 and 90, Arslan et al. (2021) noticed a decrease in their levels on post‐op Days 45 and 90. The discrepancy observed between the present study's findings and those reported by Arslan et al. (2021) can be explained by the differences in disease activity and sampling sites of the collected GCF samples. Additionally, Padial‐Molina et al. (2015) observed that periostin levels in GCF increased abruptly after surgical periodontal treatment (open flap debridement) and correlated with the healing process. The findings of the present study were consistent with those of Al‐Rihaymee and Padial‐Molina and co‐workers. The newly formed periodontal ligament and periosteum release high levels of periostin during their formation (Al‐Rihaymee and Sh Mahmood 2023). Therefore, periostin levels can increase following periodontal therapy. Following NSPT, their levels increased immediately from the early stages of inflammation, reaching peak levels upon completion of granulation tissue maturation. These findings may indicate that periostin plays a critical role in the early stages of post‐treatment ECM remodelling and contributes to fibrosis (Hamilton 2008). Their levels decreased at the middle stages of maturation (Day 30) and declined to baseline levels at the beginning of the late stage (Day 90). These stage‐dependent alterations in periostin levels suggest that periostin may reflect the biological processes underlying periodontal healing, supporting its potential as a healing biomarker.

IL‐1β plays a role in oral wound healing by controlling defence mechanisms against commensal bacteria in the oral cavity (Graves et al. 2001). IL‐1β levels in GCF were higher in the periodontitis group compared with the periodontally healthy group in the present study. Their levels were higher in the presence of periodontitis and decreased dramatically from Day 14 to Day 90. In line with previous studies, it was a predictable finding that IL‐1β levels were higher in patients with periodontitis (de Lima Oliveira et al. 2012; Sari et al. 2022). While one study showed a decrease in IL‐1β levels during the early healing period (1 week) (Gündoğar et al. 2016), other studies reported an early increase after NSPT (de Melo Soares et al. 2019; Giannopoulou et al. 2012). In the present study, although not statistically significant, IL‐1β levels increased up to Day 2 following periodontal treatment, in line with the previous studies (de Melo Soares et al. 2019; Giannopoulou et al. 2012). The subsequent reduction during the late healing phase was also in agreement with earlier reports (de Lima Oliveira et al. 2012; Romano et al. 2018). In the present study, this decrease aligned with the expected progression of wound healing and the resolution of inflammation, indicating a marked attenuation of inflammatory activity by Day 14 following NSP.

FGF‐2 plays a role in all phases of wound healing, including cell migration, differentiation and angiogenesis in the periodontal ligament (Murakami 2011). In the present study, the total amount of FGF‐2 in GCF was similar in both groups. After NSPT, their levels increased and peaked on Day 2. Although their levels remained higher than baseline on Day 90, they gradually decreased from Day 2 onwards. One study evaluated FGF‐2 levels in GCF after NSPT and found that the FGF level decreased gradually from Day 14 to Day 90 post treatment (Giannopoulou et al. 2012). The findings of the present study are in contrast with those of the previous study (Giannopoulou et al. 2012). These differences in findings may arise from variations in case definition, NSPT technique and GCF collection methods used in the previous study. The persistence of high levels of FGF‐2 from 24 h to the third month after NSPT may be related to its role in both early and late periodontal wound healing, as well as to FGF‐2's multifaceted properties.

Periodontitis was associated with increased serum IL‐1β levels, and after periodontal treatment the levels decreased gradually in the present study. These findings support the literature suggesting that local periodontal inflammation may contribute to systemic cytokine dysregulation (Nanakaly et al. 2024; Teles et al. 2025). Serum FGF‐2 and periostin levels did not differ. These results were consistent with previous studies (Balli et al. 2015; Yılmaz Şaştım et al. 2021). These findings suggest that periodontal disease and its treatment have limited systemic effects on regenerative markers, as these markers are produced locally. The concentration of regenerative biomarkers in serum may not reach levels detectable in systemic circulation during the healing phase of localised periodontal tissues.

Improvements in periodontal clinical parameters following NSPT in the present study were accompanied by changes in biomarker levels, including decreased IL‐1β levels in serum and GCF and altered periostin and FGF‐2 levels in GCF. These findings suggest that these analytes reflect different aspects of periodontal inflammation, wound healing and tissue regeneration. Assessment of local periostin levels could support the evaluation of tissue response beyond conventional clinical parameters and may contribute to more individualised maintenance protocols. Unlike inflammatory targets, which primarily reflect non‐specific disease activity, periostin is involved in ECM organisation, collagen fibrillogenesis and tissue remodelling (Padial‐Molina et al. 2015), all of which are critical to periodontal wound healing. Therefore, the increase in periostin levels following treatment may reflect ongoing healing and regenerative processes within periodontal tissues.

## 5 Conclusion

Findings of this study suggest that periostin is involved in the local tissue inflammatory process to tolerate increased inflammation and in the early healing mechanism after NSPT, serving as a regenerative biomarker independent of its serum content. Clinical findings demonstrating wound healing after NSPT, along with changes in periostin levels that parallel FGF levels, suggest that periostin may serve as a biomarker to monitor periodontal wound healing.

## Author Contributions

All authors contributed to the study conception and design. Aysegul Sarı, Alpdogan Kantarci and Oguzhan Ozcan performed the work. Aysegul Sarı performed data collection and statistical analysis. Oguzhan Ozcan and Filiz Kaçmaz performed biochemical analyses. Aysegul Sarı, Alpdogan Kantarci, Oguzhan Ozcan, and Anton Sculean wrote the first draft of the manuscript. All authors commented on previous versions of the manuscript, read and approved the final version.

## Funding

This work was supported by the Hatay Mustafa Kemal University Project Number 19.M.051.

## Ethics Statement

The study protocol was approved by the Ethics Committee for the Use of Human Subjects in Research at Hatay Mustafa Kemal University, Hatay, Turkey (Protocol No. 2019/118), and the study was conducted in accordance with the Declaration of Helsinki.

## Consent

Informed consent was obtained from all individual participants included in the study.

## Conflicts of Interest

The authors declare no conflicts of interest.

## Data Availability

The data that support the findings of this study are available on request from the corresponding author. The data are not publicly available due to privacy or ethical restrictions.
